# Extracellular vesicles in dairy cattle: research progress and prospects for practical applications

**DOI:** 10.1186/s40104-025-01242-5

**Published:** 2025-08-04

**Authors:** Nan Wang, Boqi Zhang, Juan J. Loor, Chunjin Li, Xu Zhou

**Affiliations:** 1 College of Animal Sciences, Jilin University, Changchun, 130062 China; 2 Department of Animal Sciences and Division of Nutritional Sciences, University of Illinois, Urbana, 61801 USA

**Keywords:** Extracellular vesicles, Gastrointestinal tract, Immunity, Mastitis, Metabolism

## Abstract

Intensive dairying has diminished infectious disease resistance in dairy cattle and increased the risk of disorders affecting milk quality and productive life. Development of novel health monitoring technologies, optimization of disease treatment protocols using novel biomarkers, and development of antibiotic substitutes are necessary to further enhance the productivity of dairy cattle. Extracellular vesicles (EVs) are key mediators of cellular communication and are essential for maintaining intracellular homeostasis and regulating various physiological and pathological processes. Establishing a network of mechanisms by which EVs regulate physiological processes in dairy cattle will contribute to the development of new technologies for early disease diagnosis and disease treatment. This review summarizes the molecular characterization and advances in the study of EVs in dairy cattle and focuses on the reported mechanisms of action. Prospects and limitations for the application of EVs in monitoring health status, disease treatment and assisted reproduction are discussed.

## Introduction

Dairy cattle farming occupies a key position in global livestock production and is an integral component of the food security efforts [1]. Accelerating the development of the dairy industry is important for enhancing human health and improving economic efficiency [2, 3]. During the past few decades, the increase in the scale of dairy farming, rise in milk yield by dairy cows due to genetic improvement and breeding, improvement in feeding management, optimization of the breeding environment, and progress in disease prevention and control have collectively, enhanced production efficiency [4–6].

Reducing losses due to disease and multiple stresses, increasing reproductive potential, and genetic improvement are key to improve economic benefits, including the use of high-throughput phenotyping platforms for precision livestock management in the dairy industry [7]. However, there are still bottlenecks in the development of technologies for health assessment and early detection of diseases in dairy cattle. Blood parameters are an important reference for disease diagnosis [8], but examination via blood can cause discomfort to the cattle and is time consuming and laborious. The development of non-invasive and automated monitoring techniques not only safeguards animal welfare, but also fulfills economic needs [9]. Disease monitoring in dairy cattle based on artificial intelligence (AI) and sensors has been effective in commercial settings [10], but the sensitivity and specificity of the sensors need to be further improved. For example, sensor systems based on rumination time and activity time as parameters are able to accurately monitor the health of postpartal cattle [11], but do not recognize the type of disease. Salzer et al. developed a wireless nose ring sensor system that can assess stress, disease, and critical conditions in dairy cattle via readings of heart rate, breathing rate, and oxygen saturation at the nostrils [12]. Analyzing sensor data through AI algorithms provides a reference for assessing the onset and severity of disease, but its accuracy is still sensitive to changes in temperature and motion. The identification of disease biomarkers in milk and urine is beneficial for the development of disease monitoring systems in dairy cattle. The complexity of the diseases also requires the development of more detailed and accurate diagnostic methods.

Extracellular vesicles (EVs) are released from various cell types and act as signaling structures and carriers for molecules involved in the regulation of biological processes in the organism [13]. EVs are classified into two main subtypes: (i) exosomes are small EVs (50–150 nm), which are released by the fusion of multivesicular endosomal bodies with the plasma membrane; (ii) ectosomes/microvesicles (100 nm–1 µm) arise from outward protrusions of plasma membrane [14]. In this review, we use the generic term “extracellular vesicles” according to the MISEV2023 guidelines [15]. EVs reflect the state of the original cells and the physiological condition of the organism can be monitored by analyzing changes in the concentration and content of EVs [16, 17]. Furthermore, EVs are stably present in a variety of body fluids such as breast milk, urine and semen, and have emerged as a potential biomarker for non-invasive diagnosis of disease and metabolic status [18]. By comparative analysis of miRNA profiles in milk-derived EVs from healthy and enzootic bovine leukosis (EBL) dairy cattle, Tsukada et al. identified that bta-miR-1246 and -424-5p were significantly elevated in EBL cattle. These miRNAs may facilitate tumor cell proliferation and migration by targeting tumor suppressor genes, suggesting their potential utility as biomarkers for EBL diagnosis [19].

Appropriate disease treatments are beneficial in protecting animal welfare and reducing economic losses. For infectious diseases such as mastitis, the use of antibiotics as the preferred method of treatment is problematic due to safety issues [20, 21]. Thus, finding new alternative treatments to antibiotics such as the use of traditional herbal plants, probiotics, and nutraceuticals is vital. EVs are highly targeted, cross biological membranes such as blood–brain barrier, and can avoid phagocytosis by macrophages [22]. Utilizing EVs as drug carriers can also improve drug solubility and targeted delivery for precise treatment [23, 24].

The integration of nanotechnology and AI algorithms has significantly enhanced the potential for detecting and delivering EVs in clinical applications. A recent study demonstrated that EVs can be visualized and detected from multiple angles using freeze–thaw-induced floating patterns of gold nanoparticles. The application of AI algorithms further enabled the precise identification of EVs derived from breast cancer subtypes, offering a novel tool for early breast cancer diagnosis [25]. Additionally, computer simulation will contribute to the prediction of the assembly of EV carriers, the release process of EVs in vivo and the interaction of EVs with target organs [26]. AI algorithms are capable of analyzing and organizing large-scale molecular databases. AI can analyze miRNA profiles and proteomic data in EVs to uncover the changes in cargo molecular of EVs or the mechanism of EVs uptake, facilitating health status detection and the precise delivery of EV-based drug carriers in dairy cattle [27]. Many evidences have emphasized the role of EVs in disease detection and treatment [28, 29], but the studies are mainly based on mouse models, and the application of EVs in the treatment and diagnosis of dairy cattle remains challenging. Additionally, establishing standardized protocols for sample pre-processing, as well as the production, modification, and storage of EVs, is essential to ensure the stability and reliability of EV assays and treatments.

This review summarizes the current research on EVs derived from diverse sources in dairy cattle and discusses their functions and mechanisms on metabolic processes, immune response, reproduction, lactation, gastrointestinal function, and neurodevelopment. Additionally, it explores the potential application of EVs in dairy production, including health assessment, disease diagnosis and treatment, and assisted reproduction, alongside the associated techniques. Finally, the review highlights the limitations and prospects for advancing this field.

## EVs in dairy cattle

The roles and characterizations of different origins of EVs in cattle are summarized in Table 1.

**Table 1:** Comparison of EVs from different origins in dairy cattle

Origins	Diameter	Concentration of EVs	Main outcome	References
Milk	155.1 ± 16 nm	NA	Milk-derived exosomes resist bile and enzymatic digestion	[30]
Milk	142.7 ± 2.9 nm	NA	miR-148a in milk-derived EVs from mid-lactation cows enhances *FOXP3* expression by downregulating *Dnmt3b*, thereby promoting T lymphocyte differentiation	[31]
Milk	NA	NA	bta-miR-34a in yak milk-derived EVs attenuates hypoxic stress-induced intestinal damage through hypoxia-inducible factors and apoptosis signaling	[32]
Milk	145.6 and 145.7 nm	NA	118 proteins uniquely expressed in EVs from bovine leukemia virus-infected cattle are implicated in various biological processes, including tumor cell proliferation, leukemogenesis, and tumorigenesis	[33]
Milk	106.9 nm	NA	331 proteins identified in EVs derived from Mora buffalo milk are associated with biological functions, including immunity, metabolism, and muscle development	[34]
Milk	100 nm	NA	Eight mRNAs (*CXCL8*, *FABP5*, *TMEM156*, *DEFB4A*, *VIM*, *SRGN*, *LAPTM5* and *LGALS1*) were identified as potential biomarkers for enzootic bovine leukosis	[35]
Milk	100 nm	NA	bta-miR-1246 and -424-5p were significantly elevated in milk-derived EVs from EBL cattle, suggesting their potential utility as biomarkers for EBL diagnosis	[19]
Blood	91 ± 26 nm	NA	The number of EVs derived from the plasma of fertile cows was 50% higher than that of subfertile cows	[36]
Blood	110 and 130 nm	NA	EVs carry potential protein biomarkers of fertility status, including DNAI4, HSPA4, MYOM2, WFIKKN2, MAN2B1, PXDN, IDE, AP1G1 and DAG1	[37]
Blood	100–150 nm	NA	Elevated levels of bta-miR-17-5p, -24-3p and -92a in blood EVs from EBL may contribute to disease progression by promoting apoptosis and neoplasia	[38]
Blood	30–200 nm	1 × 10^8^ particles/mL	EVs derived from high-fertility cows downregulated the expression of prostaglandin synthases and proinflammatory cytokine in bovine endometrial stromal cells	[39]
Urine	NA	NA	The decrease in AQP2-bearing EVs in pregnant heifers may be attributed to both diminished EV release into the urine and reduced AQP2 expression in the kidney	[40]
Rumen	157.4 nm	1 × 10^10^ particles/mL	Ruminally-derived EVs enhanced pathogen resistance and growth by upregulating the expression of immune genes and developmental metabolic genes, including *irg-5*, *fbxa-116* and *pas-3*	[41]
Parasite	30–100 nm	NA	EV proteins derived from the rumen fluke *Calicophoron daubneyi* increased ruminal bacterial diversity by facilitating the survival of specific bacterial species	[42]
Bacterium	49 nm	20 μg/mL	*Fibrobacter succinogenes* enhances rumen digestive efficiency by releasing outer membrane vesicles enriched with carbohydrate-active enzymes, facilitating the degradation of cellulose	[43]
Follicular fluid	142 and 128 nm	NA	EVs can stimulate cumulus-oocyte complex expansion by increasing the expression of *PTGS2*, *PTX3* and *TNFAISP6*	[44]
Follicular fluid	NA	NA	EVs can exert regulation of oocyte maturation process by altering *IGFBP2*, *CDCA8* and *STAT3* expression	[45]
Follicular fluid	70–400 nm	NA	Exosomal miR-34c promoted cell proliferation by targeting cell cycle regulators, enhancing oocyte competence acquisition and embryo quality	[46]
Follicular fluid	124.8 ± 2.4 and 147.1 ± 5.6 nm	NA	bta-miR-431, -136, -370, -376e, and -411c-3p were exclusive expressed in follicular fluid EVs from cows with consistently negative energy balance	[47]
Follicular fluid	30–200 nm	NA	Supplementation of EVs derived from 3–6 mm follicles enhanced DNA methylation and hydroxymethylation levels, increasing the blastocyst rate	[48]
Oviduct	NA	NA	The abundance of proteins (AQP5, HSPA8, and MIF) related to reproductive function was higher in post-ovulatory EVs than in pre-ovulatory	[49]
Oviduct fluid	NA	0.05 mg of proteins/mL	EVs significantly changed the phospholipid composition before and after blastocoel expansion, and the total cell number in expanded blastocysts tended to increase during bovine embryo culture in vitro	[50]
Oviduct	30–200 nm	NA	Nine miRNAs in oviduct-derived EVs were differentially abundant between stages of the estrous cycle, eight of them were increased progressively between stage 1 and stage 3	[51]
Uterine	30–200 nm	NA	14 miRNAs were differentially abundant in EVs of uterine fluid between different stages	[51]
Uterine	154.9 ± 20.8 and 236.7 ± 22.5 nm	2.4 × 10^8^ particles/mL	Endometrial-derived EVs from early luteal-phase cows induced interferon tau expression and increased embryo diameter	[52]
Uterine	50–150 nm	3 × 10^5^ EVs/mL	Supplementation of uterus-derived EVs reduced apoptosis and improved the inner cell mass/trophectoderm cell ratio	[53]
Amniotic cells	275 ± 8.4 nm	100 × 10^6^ EVs/mL	The addition of amniotic EVs improved the hatching and pregnancy percentages of in vitro bovine embryos	[54]
Amniotic cells	258 ± 55 nm	100 × 10^6^ EVs/mL	Amniotic EV reduced apoptosis in embryos by down-regulating the *BAX* gene and up-regulating the *GPX1* gene	[55]
Embryo	60–150 nm	NA	Embryo-derived EVs significantly improved blastocyst formation, total cell number, and calving rate	[56]
Semen	190.10 ± 11.35 nm	NA	Heat stress influences testicular and epididymal function by altering the miRNA composition of seminal plasma EVs	[57]
Semen	152.2 ± 1.8 nm and 146.4 ± 1.1 nm	NA	bta-miR-195 was expressed approximately 80% higher in high-fertile bulls compared to the low-fertile group, suggesting its potential as an indicator for fertility detection	[58]

NA, Not applicable. Concentration of EVs, Concentration of EVs used in the treatment

### Milk

Milk is a rich source of immunoglobulins, vitamins, growth factors, bioactive molecules, and milk-derived EVs all of which are critical for promoting growth and development of calves [59]. Bovine milk-derived EVs contain high amounts of EVs from different cellular sources such as adipocytes and immune cells in the mammary gland [60]. Interestingly, the encapsulation of EVs in bovine milk ensures the stability of their contents during gastrointestinal digestion and facilitates their transport to various tissues, therefore allowing for the efficient transfer of maternal messages to the neonate [30]. Some miRNAs in milk-derived EVs also influence innate and adaptive immunity through epigenetic regulation. For example, miR-148a in milk-derived EVs from mid-lactation cows enhances FOXP3 expression by downregulating *Dnmt3b*, which drives lymphocyte differentiation into anti-inflammatory regulatory T cells [31]. bta-miR-34a in yak milk-derived EVs also was reported to exert a protective role on the intestinal barrier by reducing intestinal damage that hypoxic stress causes via hypoxia-inducible factors and apoptosis signaling [32]. To investigate the physiological and immunological properties of proteins in milk-derived EVs, Rahman et al. analyzed the proteome of milk-derived EVs from bovine leukemia virus (BLV)-infected and uninfected cattle. The proteomic analysis identified 118 proteins uniquely expressed in EVs from BLV-infected cattle. These proteins are likely implicated in various biological processes, including tumor cell proliferation, leukemogenesis, cancers, and tumor formation [33]. Proteomic analysis of Murrah buffalo milk-derived EVs also showed that 331 proteins were identified with biological functions related to immunity, metabolism and muscle development [34]. In addition, Hiraoka et al. reported significantly elevated levels of eight mRNAs (*CXCL8*, *FABP5*, *TMEM156*, *DEFB4A*, *VIM*, *SRGN*, *LAPTM5* and *LGALS1*) in milk-derived EVs from BLV-infected cattle compared to uninfected cattle. These mRNAs are associated with cellular activity, cell migration and invasion, immune regulation, and cancer metastasis, and were identified as potential biomarkers for EBL [35]. Another study also showed that the significant elevation of miR-1246 and -424-5p in milk-derived EVs from BLV-infected cows may promote the proliferation and migration of tumour cells [19].

### Blood

Blood EVs, produced by cells and then released into the systemic circulation, contain important information specific to the cell of origin and are reflective of the cellular microenvironment. For example, by comparing blood EV profiles in the plasma of two strains of dairy cows with divergent fertility phenotypes (New Zealand Holstein–Friesian strain and North American Holstein–Friesian strain), Mitchell et al. observed a greater concentration of EVs in cows with fertile phenotypes [36]. Using plasma EV proteomes and reproductive outcomes in primiparous and peripubertal cows, Turner et al. identified 9 proteins associated with fecundity, DNAI4, HSPA4, MYOM2, WFIKKN2, MAN2B1, PXDN, IDE, AP1G1 and DAG1 [37]. Takada et al. observed elevated levels of bta-miR-17-5p, -24-3p and -92a in blood EVs from EBL cattle compared to the uninfected cattle. These miRNAs may contribute to disease progression by promoting apoptosis and neoplasia [38] and could serve as potential biomarkers for the diagnosis of EBL. Abeysinghe et al. studied the effects of EVs enriched in plasma from cows of different fertility on bovine endometrial epithelial and stromal cells and observed that EVs in cows of high fertility caused downregulation of prostaglandin synthases and proinflammatory cytokine in the cells [39]. Thus, they concluded that blood EVs may affect reproductive outcomes by modulating the receptivity of endometrium.

### Urine

Urine is more available in a non-invasive manner than other body fluids, thus, urine-derived EVs can serve as mediators of signals in the urinary system and may be a source of biomarkers for recognizing pathological states. For example, aquaporin-2 (AQP2) was identified as a protein associated with renal or blood pressure regulation disorders in studies of urinary EVs in humans and rats [61, 62]. The AQPs accumulate in the female reproductive tract of rats in late-pregnancy, and the mRNA level of *Aqp5* in the blood is significantly increased during the last day of pregnancy, which provides a reference for predicting the initiation of delivery [63]. A study reported that the level of urinary AQP2 in human was increased during pregnancy to enhance V2 (arginine vasopressin receptor 2) receptor-mediated water retention [64, 65]. Sinlapadeelerdkul et al. collected urine through urethral catheterization in the morning from pregnant cows in the first, second and last trimester, and isolated EVs from urine and compare the levels of AQP2-bearing EVs. They found that the level of AQP2 in urine-derived EVs from pregnant cows was reduced and became lower as pregnancy progressed, which may be due to decreased release of EVs into the urine and decreased expression of AQP2 in the kidney [40]. The EVs produced by the reproductive tracts may be released into the urine, thus, urinary EVs may contain information about the reproductive system [66]. These features may provide a theoretical basis for the development of novel markers in the detection of pregnancy and pregnancy disorders.

### Gastrointestinal tract

Ruminal microbial fermentation can help meet upwards of 70% of daily energy requirements, thus, is a key factor in the feed efficiency of dairy cattle [67]. Microorganisms of the lower intestinal region also play an important role in the development of the immune system of dairy cattle [68]. Because EVs are important mediators of the regulation of microbe-host interactions [69], it is likely that abundance and composition of EVs in the gastrointestinal tract is critical for maintaining health and productive performance.

Choi et al. used the model organism *Caenorhabditis elegans* to study the genetic effects of EVs on the host and demonstrated that ruminally-derived EVs promoted pathogen resistance and growth by upregulating the expression of immune genes and developmental metabolic genes (*irg-5*, *fbxa-116* and *pas-3*) [41]. Allen et al. demonstrated that EVs released by the rumen Fluke *Calicophoron daubneyi* increased ruminal bacterial diversity, in part due to the immunomodulatory activity of EV proteins, which promoted the survival of certain bacterial species [42]. Additionally, key cellulolytic organisms such as *Fibrobacter succinogenes* enhance rumen digestive efficiency by releasing outer membrane vesicles enriched with carbohydrate-active enzymes, facilitating degrade cellulose [43]. Together, these findings highlight the role of EVs and their potential use as biologics in the dairy industry. By having a direct effect on ruminal bacteria and the host, EVs could be valuable in efforts to improve feed utilization efficiency in the future.

### Others

Other biological sources of EVs include follicular fluid, oviduct, uterine fluid, amniotic fluid, vaginal mucus, semen, saliva, ascites, bile, cerebrospinal fluid, and feces [70, 71]. Bovine follicular fluid-derived EVs can stimulate cumulus-oocyte complex expansion by increasing the expression of *PTGS2*, *PTX3* and *TNFAISP6* [44] and exert regulation of oocyte maturation process by altering *IGFBP2*, *CDCA8* and *STAT3* expression [45]. Benedetti et al. reported that miR-34c in follicular fluid-derived EVs promoted cell proliferation by targeting cell cycle regulators, thereby enhancing oocyte competence acquisition and embryo quality [46]. In a separate study, post-calving energy balance was linked to changes in miRNA profiles within follicular fluid EVs. Specifically, bta-miR-431, -136, -370, -376e, and -411c-3p were exclusive expressed in cows with consistently negative energy balance [47]. da Silveira et al. demonstrated that supplementing embryo culture media with EVs derived from follicular fluid from 3–6 mm bovine ovarian follicles upregulated genes involved in epigenetic modification (*DNMT3A*) and cell cycle (*CDH1* and *REST*), resulting in enhanced DNA methylation and hydroxymethylation levels, ultimately increasing the blastocyst rate [48].

The EVs from the oviduct deliver key molecules to gametes and embryos, playing an important role in gamete fertilization and early embryo development [72]. By analyzing the transcriptome and proteome of bovine oviduct-derived EVs at different stages of the estrous cycle, Alminana et al. demonstrated that the contents of oviduct-derived EVs were influenced by estrogen and progesterone concentrations, and that the abundance of proteins (AQP5, HSPA8, and MIF) related to reproductive function was higher in post-ovulatory EVs than in pre-ovulatory [49]. Banliat et al. showed that oviduct-derived EVs treatment significantly changed the phospholipid composition before and after blastocoel expansion, and the total cell number in expanded blastocysts tended to increase during bovine embryo culture in vitro [50]. Hamdi et al. identified nine miRNAs in oviduct-derived EVs that were differentially abundant between stages of the estrous cycle, eight of them were increased progressively between stage 1 and stage 3; 14 miRNAs were differentially abundant in EVs of uterine fluid between different stages [51]. The functional enrichment analysis revealed that these miRNAs were involved in the regulation of the fallopian tube and uterus, as well as pregnancy and embryonic development.

Aguilera et al. demonstrated that endometrial-derived EVs from early luteal-phase cows induced interferon tau (IFNT) expression and increased embryo diameter [52]. Qiao et al. demonstrated that supplementing in vitro embryo cultures with bovine uterine EVs during the early luteal phase reduced apoptosis and improved the inner cell mass/trophectoderm cell ratio. This was mediated by the downregulation of *HSP70*, *BAX* and *BIP*, along with the up-regulation of IFNT and acrogranin [53].

Lange-Consiglio et al. reported that the addition of amniotic EVs during bovine embryo production in vitro could alter the expressions of miR-130a and miR-181b that regulate blastocyst development and embryo implantation, thus affecting the quality of embryo development and implantation potential [54]. Perrini et al. demonstrated that supplementing bovine oviducal fluid on d 5 post-fertilization with endometrial or amniotic EVs reduced apoptosis in embryos. This was achieved by down-regulating the *BAX* and up-regulating the *GPX1* genes, leading to higher blastocyst production rate and inner cell mass [55]. Similarly, Qu et al. reported that adding bovine embryo-derived EVs to embryo media significantly improved blastocyst formation, quality (evidenced by increased *OCT-4* mRNA levels and total cell number), and calving rate [56].

The EVs released from epididymis epithelial cells are important communication mediators in regulating the final stages of spermatogenesis [73]. Heat stress has been demonstrated to downregulate miR-34b, -34c, -146a, and -15a in bovine seminal plasma EVs, which reduces sperm quality by impairing mitochondrial function, triggering pro-inflammatory responses, and activating DNA damage [57]. Chauhan et al. identified 41 miRNAs with significant expression differences in seminal plasma EVs between high-fertile and low-fertile bulls. Among these, bta-miR-195 was expressed approximately 80% higher in high-fertile bulls compared to the low-fertile group. Gene Ontology (GO) and Kyoto Encyclopedia of Genes and Genomes (KEGG) pathway analysis revealed that the targets of bta-miR-195 are associated with sperm motility and acrosome reaction, suggesting its potential as an indicator for fertility detection in Sahiwal bulls [58]. Although data are only available for establishing the function of locally produced EVs in dairy cattle, there is need to generate information on the interactions among EVs produced by different organs.

## Biological functions of EVs

**Fig. 1:**
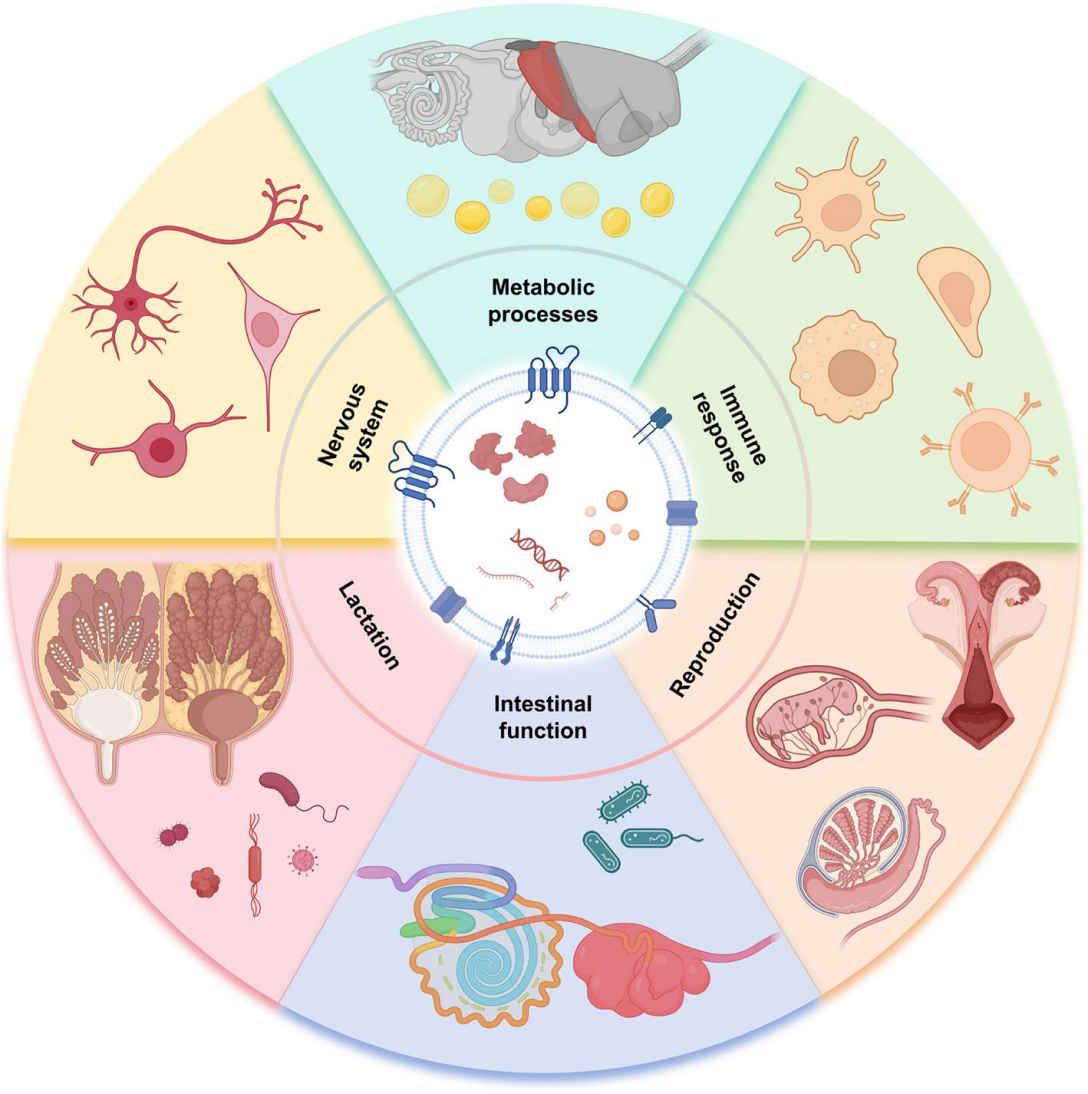
Role of extracellular vesicles on physiological processes in animals. Extracellular vesicles regulate health by affecting metabolic processes (liver and adipose tissue), immune response (macrophages, T cells, B cells, monocytes and neutrophils), reproduction (female reproductive system, male reproductive system and fetal development), lactation (mammary gland development, milk formation and mastitis), gastrointestinal function (intestinal barrier, intestinal motility, intestinal immunity), and the nervous system (axon growth, astrocytes and microglia)

The EVs are secreted by a variety of cell types and travel among organismal tissues and cells, exchanging biologically active substances and coordinating signaling (Fig. 1). In doing so, EVs can impact metabolism, immune response, reproduction, lactation, and other important physiological functions discussed below [13].

### Metabolism

There is increasing evidence that EVs are key regulators of metabolism [41, 47, 74]. For instance, Mazzarella et al. found that bta-miR-1193 and -18a, specifically expressed in bovine oviduct-derived EVs from luteal-phase heifers, regulate lipid metabolism by targeting *ACSL3*, *PLIN2*, and *PPARGC1B*. Additionally, they identified 28 miRNAs specifically present in uterine-derived EVs, which are involved in lipid uptake and transport (*CD36*, *LDLR*, and *FABP3*), lipogenesis (*ACACA* and *PPARGC1B*), and lipid accumulation (*PLIN2*) [75]. Another study also demonstrated that adding oviduct and uterine fluid-derived EVs from luteal-phase heifers into the sequential culture system of bovine embryos influenced lipid metabolism in blastocyst by decreasing *LDLR*, *PARGC1B*, *FASN* and *PNPLA2* expression and restoring phosphorylated hormone-sensitive lipase activity [76]. *Staphylococcus aureus* (*S. aureus*) EVs induce apoptosis of bovine mammary epithelial cells by up-regulating *BAX* and down-regulating *BCL2*, and block mitochondrial degradation by disrupting the acidic environment of lysosomes [77]. Ying et al. found that treatment with follicular fluid-derived EVs from bovine small follicles (3–5 mm) promoted synthesis of progesterone and androstenedione in ovarian cortical stromal cells by upregulating *CYP17A1*, *CYP11A1*, and *3β-HSD* [78]. By proteomic analysis of milk-derived EVs, Couse et al. identified an abundance of EV proteins associated with molecular processing functions, including NME2 (involved in guanosine triphosphate synthesis), IDH1 (isocitrate dehydrogenase), and PLIN2 (a lipid droplet protein) [79]. Together, these studies underscored a role for EVs in the detection and treatment of metabolic diseases.

### Immune response

EVs are carriers of molecules that can transport cargo to many organs and modulate the immune response [80]. For example, EVs isolated from the culture medium of d 5 in vitro embryos could induce down-regulation of *UNC13D* and *ARHGEF2*, genes associated with innate immune regulation and antigen presentation, and the up-regulation of *ISG15* and *OAS1*, genes associated with antiviral and immune response in bovine oviductal epithelial cells [81]. These findings suggest a potential role of EVs in mediating maternal–fetal crosstalk. Alves et al. observed the down-regulation of miR-146a in semen-derived EVs from heat-stressed bulls. Low levels of miR-146a were associated with increased inflammatory signaling, potentially triggering a pro-inflammatory response in the testis of heat-stressed bulls [57]. Mecocci et al. also revealed that milk-derived EVs are enriched with amino acids, group B vitamins and nucleotides, which play a crucial role in modulating the immune response. Specifically, vitamin B_9_ supports the maintenance of regulatory T cells, arginine facilitates the production of bacteriostatic NO, and methionine contributes to intestinal mucosal homeostasis. These components collectively aid in restoring intestinal damage and maintaining the integrity of the intestinal barrier [82].

The study showed that circulating EVs derived from low-fertility heifers activated the inflammatory response in bovine endometrial epithelial cells (EECs) by up-regulating *IL-1α* and *IL-8* while down-regulating *IL-4*. Additionally, these EVs inhibited blastocyst adhesion to the endometrium by down-regulating *CXCL9* and *CXCL10* in bovine endometrial stromal cells, ultimately impairing the establishment of pregnancy [83]. Nakamura et al. demonstrated that EVs derived from uterus of pregnant ewe on d 17 upregulated the TNF signaling pathway in bovine endometrial cells, promoting inflammatory responses. These responses play a regulatory role in uterine tolerance and embryo implantation [84]. By proteomic analysis of EVs derived from uterine fluid in cows with clinical or subclinical endometritis, Piibor et al. identified 15 immune-related pathways, including leukocyte proliferation and response to pathogens [85]. Another study employed LPS-injured bovine EECs as an in vitro model of endometritis. It demonstrated that reduced miR-331 levels in EVs derived from these cells promoted NF-κB activation via the Notch/IKK pathway, triggering excessive pro-inflammatory responses in macrophages [86]. These findings advance the understanding of uterine-derived EVs and propose molecular targets for the treatment of bovine uterine disease.

Saenz et al. observed that miRNAs linked to inflammatory response were upregulated in milk-derived EVs from cows with subclinical mastitis compared to healthy cows. Furthermore, enrichment analysis of 48 miRNA-targeted genes revealed significant alterations in KEGG pathways, including Toll-like receptor signaling pathway, NF-κB signaling pathway, and cytokine receptor interactions [87]. A study also demonstrated the enrichment of immune-related miRNAs in milk-derived EVs from mid-lactation period cows, including miR-148 (which drives T-lymphocyte differentiation), let-7 (which regulates NF-κB signaling pathway) and miR-21 (a key mediator of macrophage anti-inflammatory response) [31]. Chen et al. developed an in vitro model of selenium-deficient and selenium-control bovine mammary epithelial cell lines (MAC-T) from Holstein cows and isolated their EVs, which were added to the culture medium of normal MAC-T. The study revealed that EVs derived from selenium-deficient MAC-T cells significantly up-regulated the expression of inflammatory factors TNF-α, IL-6, and IL-8 in MAC-T cell. This effect was attributed to the differential miRNA profiles in the selenium-deficient MAC-T-derived EVs, which activated the inflammatory response by suppressing PI3K-AKT-mTOR signaling pathway [88]. In addition, *S. aureus* is one of the most prevalent microorganisms responsible for mastitis in dairy cows. EVs isolated from *S. aureus*, which contain several pathogenic proteins (alpha-hemolysin, Enolase, Sortase A) significantly up-regulated pro-inflammatory genes such as *CXCL1*, *IL-6*, *iNOS*, and *TNF* in bovine monocyte-derived macrophages, and suppressed host defenses and promoted bacterial colonization [89]. Zhu et al. identified 325 upregulated proteins (e.g., integrin alpha-6, complement 3, and recombinant desmoyokin) in *S. aureus*-infected MAC-T-derived exosomes compared to controls. Enrichment analysis showed that these differential proteins were mainly associated with innate immunity, extracellular matrix-receptor interaction and phagosomal pathways [90]. These findings provide novel insights into the role of EVs in the pathogenesis of mastitis in dairy cows. The regulatory effects of EVs on immune responses also imply their potential as therapeutic agents for mastitis. For instance, Liang et al. demonstrated that milk-derived EVs mitigated *Klebsiella pneumoniae*-induced oxidative stress and ferroptosis in bovine mammary epithelial cells by up-regulating *GPX4* and *NRF2*, thereby alleviating mammary tissue inflammation [91]. Gillan et al. compared EVs from *Theileria annulata*-infected and uninfected bovine lymphosarcoma cell lines. They found that *Theileria annulata* upregulated infection-associated proteins and downregulated bta-miR-181a (which promotes aberrant cell proliferation by targeting *ICAM-1*) in EVs. These findings enhanced the understanding on the infectious mechanisms of *Theileria annulata* [92].

In the future, it would be necessary to study the immune response regulated by EVs in more depth, to characterize the changes of EV contents in different states. This type of studies would allow identification of key molecules that play a biological function in EVs. Such information is important for formulating an effective plan to use EVs to achieve immunomodulatory control.

### Reproduction

There is growing evidence that EVs are essential for the establishment of connections between reproductive organs, regulating processes like gamete development, pregnancy maintenance, and embryo development [93, 94]. For instance, Kusama et al. demonstrated that peri-implantation bovine uterus-derived EVs contained IFNT, a pregnancy-recognizing protein. These EVs regulated the pre-implantation endometrial environment by upregulating apoptosis-associated genes (*TNF-α*, *CASP3*, *BAX* and *TP53*) and adhesion molecules (*VCAM1*) in EECs [95]. Alminana et al. identified OVGP1, HSP90, HSPA8, HSP70, Gelsolin, and Ezrin in oviduct-derived EVs from dairy cows via proteomics, all of which can enhance mammalian sperm survival in vitro. Further experiments demonstrated that oviduct-derived EVs can be taken up by the embryo to improve its development [96]. Another study revealed that EVs derived from oviductal fluid of heifers in the early luteal phase exhibited elevated bta-miR-148b level. miR-148b regulated cell proliferation and differentiation by targeting SMAD5, thus influencing embryo quality [97]. Fan et al. found that EVs derived from bovine blastocysts contain a specific tRNA fragment (tDR-14:32-Glu-CTC-1) that regulates preimplantation embryonic development by participating in actin assembly [98].

Biase et al. found that uterine fluid-derived EVs from artificially inseminated pregnant heifers had higher abundance of bta-mir-17, bta-mir-7-3, MIR7-1, and MIR18A compared to the transfer of in vitro-produced blastocysts, and that these miRNAs are involved in the regulation of trophectodermal health, endometrial receptivity, and the pregnancy establishment, resulting in an impact on pregnancy outcomes [99]. Ichikawa et al. identified eight miRNAs that were highly expressed in EVs derived from uterine fluid of low-fertility cows that failed to conceive in three consecutive inseminations. They found that these miRNAs inhibited embryonic development and pregnancy recognition by altering MAPK signaling and suppressing *IFNT* gene expression [100]. Wang et al. demonstrated that reduced miR-218 expression in EVs derived from endometrial epithelium of cows with endometritis impaired trophoblast cell migration, invasion, and differentiation by inhibiting the Wnt signaling pathway [101]. Another study revealed that miR-218 expression was significantly reduced in EVs derived from uterine tissue of endometritic cows and from LPS-treated EECs. miR-218 mimics reduced LPS-induced pro-inflammatory factors (*TNF-α*, *IL-6*, and *IL-1β*) and chemokines (*MIP-1α* and *MIP-1β*) in EECs, thus the reduction of miR-218 in EVs disrupted the immune homeostasis in the uterus [102]. These findings suggest that miRNA expression pattern in EVs may serve as candidate biomarkers for fertility assessment and disease identification in dairy cattle.

Lopera-Vasquez et al. demonstrated that the addition of oviduct-derived EVs to bovine embryos cultured in vitro improved development by increasing cryotolerance and the expression of genes related to metabolism and epigenetics [103]. Mitchell et al. demonstrated that milk-derived EVs loaded with miR-143 could improve pregnancy outcomes by inhibiting cyclooxygenase and reducing prostaglandin E_2_ production in bovine endometrial cells [104]. Together, available data suggest that EVs may play a role in improving assisted reproductive technologies.

### Lactation

Using proteomic analysis of milk-derived EVs from late-lactating cows, Rahman et al. identified an enrichment of RAB27B protein involved in exosome release, and speculated that milk-derived EVs may be associated with mammary gland degeneration and reduced milk production in late lactation [105]. Zhang et al. characterized the protein composition of bovine mammary epithelial cell-derived exosomes and cross-compared them with the proteome of milk-derived EVs in the milk-derived EVs proteomics database (http://exocarta.org), and identified 77 proteins in common [106]. GO analysis and KEGG pathway analysis revealed that these proteins have binding and catalytic functions and are involved in the milk biosynthesis-related PI3K-Akt, PPAR, and Jak2-STAT5 pathways, among which proteins such as ANXA2, ArhGAP29, and BTN1A1 have been reported to be associated with functions during lactation.

Lin et al. reported that luminal mammary epithelial cells treated with EVs derived from mesenchymal mammary epithelial cells formed mammary glands after being transplanted into fat pads, indicating that lipid rafts in EVs contain molecules that can initiate and sustain mammogenesis [107]. In addition, an in vitro study demonstrated that knockdown of Rab27a/b (proteins that control exosome secretion) inhibited formation of mammary epithelial cell polarity by impairing EV secretion, and that EVs may induce the establishment of polarity by selectively removing cellular components. The enrichment of annexin in apical EVs also implies that EVs may be involved in epithelial polarization and duct formation by promoting phagocytosis of apoptotic cells [108]. Together, these data indicate that EVs mediate cellular communication during mammary gland development and lactation. As such, more in-depth studies on the role of EVs in the context of milk production capacity and mammary gland health in dairy cows could be valuable.

### Gastrointestinal function

There is evidence for EVs as key mediators in the regulation of gastrointestinal tract function and host health in cows, goats, mice and humans [109]. For example, Liu et al. compared the miRNA expression profiles of EVs derived from colostrum (d 3) and mature milk (d 30), identifying 36 miRNAs among the top 40 most abundance that were associated with intestinal barrier integrity. Further examination demonstrated that EVs from colostrum and mature milk reversed LPS-induced dysregulation of genes related to apoptosis (*Caspase-3*, *p53*, *Bcl2*, and *Bax*), pro-inflammatory factors (*IL-1β*, *IL-6*, and *TNF-α*), and intestinal barrier-related genes (*Tjp1*, *Ocln*, and *Cldn1*) in intestinal epithelial cells. These findings indicate that colostrum-derived EVs exhibit a protective effect on the intestinal barrier and function [110]. Gao et al. characterized the proteomes of milk-derived EVs from yaks and Holstein cows during the mid-lactation period, and found that APOH and CD46 levels were significantly higher in yaks than in cows. Additionally, in vitro experiments demonstrated that yak milk-derived EVs more effectively alleviated LPS-induced intestinal inflammation and improved the intestinal barrier function by inhibition of the PI3K-AKT/C3 pathway [32]. The milk-derived EVs also increased beneficial microorganisms such as *Akkermansia* and *Turicibacter*, and levels of serum anti-inflammatory factors, modulating the intestinal immune network [111]. These findings indicate that milk-derived EVs may play a significant role in mitigating intestinal inflammation and enhancing intestinal barrier integrity. Criscitiello et al. isolated EVs from the serum of 5-month-old Holstein steers and characterized their deiminated proteins. Notably, they identified “intestinal immune network for IgA production” as identified for proteins common in serum EVs [112].

Gut microbiota is an important factor affecting gut health [113], and bacterial EVs may be one of the pathways whereby gut microorganisms regulate the intestinal microenvironment, intestinal barrier and host health. The bacterial EVs can serve as carriers of signaling molecules such as nucleic acids, toxins and proteins secreted by gut bacteria, and can effectively help colonize and protect bacteria from antibiotics and maintain gut microenvironmental homeostasis [114]. *Calicophoron daubneyi* is a major species of rumen fluke infecting ruminants. Allen et al. successfully isolated EVs released by *Calicophoron daubneyi* and identified 378 soluble proteins. Among these proteins, Sigma-class glutathione S-transferase and cathepsin L and B proteases exhibit immunomodulatory effects on the host and regulate microbial species diversity by influencing antimicrobial activity [42]. In addition, serotonin is an important signaling molecule that regulates intestinal secretion and motility [115]. Yaghoubfar et al. reported that human epithelial colorectal adenocarcinoma cells treated with bacterial EVs secreted by *Akkermansia muciniphila* (*A. muciniphila*) and *Faecalibacterium prausnitzii* significantly increased the expression of serotonin system-related genes [116]. Arntzen et al. demonstrated that the EVs secreted by *Fibrobacter succinogenes* are enriched with carbohydrate-active enzymes, which facilitate the efficient degradation of diverse plant polysaccharides [43]. Another study reported that the EVs of *Bacteroides thetaiotaomicron*, a commensal bacteria of the gut, improve nutrient absorption by translocating InsP6 phosphatase and eliminating excess InsP6 [117]. Research on microbial-derived EVs will deepen our understanding of microbial function and provide potential strategies to improve gastrointestinal health, digestion, and nutrient absorption.

### Nervous system

EVs are important players in signaling connections and material transport within the nervous system [118]. For example, Frohlich et al. reported that EVs secreted by oligodendrocytes can affect neuronal physiology by translocation of biologically active molecules (catalase, Hsc/Hsp70, SOD1, *Plp* mRNA), activation of signaling pathways (Erk, JNK, and Akt), and reduction of neuronal dystroglycan expression [119]. *Histophilus somni* (*H. somni*), an opportunistic pathogen, is responsible for neurological, reproductive, and respiratory diseases in cattle. Hellenbrand et al. found that bovine macrophages required stimulation of *H. somni* EVs to induce neutrophil extracellular trap formation, which subsequently captured and eliminated *H. somni* [120]. Rivera et al. reported that EVs derived from *H. somni*-infected bovine brain endothelial cells enhanced the procoagulant activity of bovine peripheral blood polymorphonuclear neutrophils, leading to thrombosis within brain microvessels and consequently the onset of bovine central nervous system disease [121]. Bacterial EVs provide novel insights into host-bacteria interactions and demonstrate significant potential as vaccine candidates for pathogenic bacteria. In addition, Slow Progressive Hidden INfections of variable latency (SPHINX) DNA sequences exhibited significantly enriched in the tissues of cows diagnosed with bovine spongiform encephalopathy. Dhurve et al. demonstrated that EVs derived from *Acinetobacter baumannii *strains DS002 can traverse the blood–brain barrier, delivering circular DNA molecules (SPHINX) to the central nervous system [122]. This study provides novel insights into the role of EVs in facilitating the lateral mobility of bacterial DNA.

## Application of EVs in dairy production

As outlined in the sections above, the EVs are involved in the regulation of key physiological processes in the body, thus, are of great potential for clinical applications such as health status monitoring, targeted therapies, nutritional management and assisted reproduction in dairy cattle (Table 2).

**Table 2:** Application of EVs in dairy production

**Application**	**Process**	**Source**	**Species**	**Main outcome**	**References**
Monitoring of health status	Estrus detection	Milk	Cow	133 differentially expressed exosomal miRNAs were identified in estrous cow milk vs. non-estrous cow milk	[123]
	Mastitis diagnosis	Milk	Cow	Chronic subclinical mastitis induces changes in milk EVs miRNA cargo. bta-miR-223-3p is the most up-regulated miRNA	[87, 124]
	Mastitis diagnosis	Milk	Cow	miR-375 was significantly decreased in milk-derived EVs from cows with mastitis compared to healthy cows	[125]
	Early pregnancy diagnosis	Placenta	Cow	bta-miR-499 was significantly elevated in the plasma of placenta-derived EVs from cows during early pregnancy	[126]
	Pregnancy status	Blood	Cow	The relative abundances of miR-25, -16b and -3596 were significantly increased in cows exhibited embryonic mortality compared to pregnant cows	[127]
	Artificial breeding	Semen	Bull	The level of bta-miR-195 in seminal plasma was related to fertility status and can be used as a potential marker for selecting high-fertility bulls in artificial breeding	[58]
	Metabolic status	Follicular fluid	Cow	The expression of miRNAs is elevated in the EVs of positive energy-balanced cows and decreased in the EVs of negative energy-balanced cows	[47]
	Endometritis diagnosis	Uterine fluid	Cow	HTRA1 as a potential biomarker for cows with subclinical endometritis	[85]
	Biomarker of fertility	Blood	Cow	Quantification of EV proteins associated with fertility phenotypes by bioinformatics pipelines	[37]
Treatment and prevention of diseases	Prevention of subclinical mastitis	Umbilical cord blood mesenchymal stem cells	Cow	The administration of EVs prepared from bovine umbilical cord blood mesenchymal stem cells alleviated subclinical mastitis in dairy cows	[128]
	Protection of embryonic development	Endometrial epithelial cells	Cow	The decreased miR-218 in endometrial epithelial-derived EVs led to the release of sFRP2 inhibition of placental trophoblasts, causing the disruption of cow embryo development under conditions of endometrial inflammation	[101]
	Treatment of calf diarrhea	Colostrum	Cow	Pretreatment with colostrum-derived EVs exhibited anti-inflammatory via downregulating *TLR5*, *STING*, and *CGAS* in cells, as well as reducing adhesion of *Escherichia coli*	[129]
	Treatment of mastitis	Milk	Cow	EVs derived from colostrum, mature milk and mastitis milk alleviated *Klebsiella pneumoniae*-induced oxidative stress and ferroptosis in bovine mammary epithelial cells	[91]
	Drug carrier	Milk	Cow	The EVs loaded with isobavachalcone and polymyxin B significantly increase antimicrobial activity and accelerate wound healing	[130]
	Drug carrier	Milk	Cow	The EVs, as drug carriers, significantly increase the solubility and antimicrobial efficiency of α-mangostin	[131]
	Vaccine	*Staphylococcus aureus*	Cow	*Staphylococcus aureus*-derived EVs are enriched with toxins and cell surface proteins that elicit immune responses, highlighting their potential as vaccine candidates	[89]
Assisted reproduction	Protection of ovarian function	Mesenchymal stromal cells	Cow	Intraovarian injection of mesenchymal stromal cell-derived exosomes mitigates ovarian damage induced by ovum pick-up	[132]
	Pregnancy establishment and maintenance	Milk	Cow	Milk-derived EVs loaded with miR-143 reduced prostaglandin E_2_ production in bovine EECs by suppressing cyclooxygenase, thereby supporting pregnancy establishment and maintenance	[104]
	Protection of oocytes	Granulosa cell	Bovine	EVs isolated from bovine granulosa cell culture medium mitigated the adverse effects of heat stress on oocytes	[133]
	Improvement of embryo quality	Follicular fluid	Bovine	bta-miR-34c in follicular fluid-derived EVs from receptive oocytes improves blastocyst quality during in vitro maturation	[46]
	Enhancement of sperm function	Follicular fluid	Bull	EVs derived from follicular fluid of bovine large, medium and small follicles significantly improved sperm viability, capacitation and acrosome reaction	[134]
	Prediction of superstimulatory response	Blood	Heifer	miR-199a-3p in EVs was upregulated after the superstimulatory response, whereas miR-17-5p and miR-182 were downregulated	[135]
	Enhancement of embryo quality	Uterus	Bovine	EVs derived from the bovine luteal phase uterus increased blastocyst formation, hatching rate, blastocyst quality and structural integrity	[53]
		Oviduct	Cow	miR-148b in oviductal fluid-derived EVs can activate SMAD5-dependent BMP signaling to regulate cell proliferation and differentiation in embryos	[97]
	Improvement of sperm cryostability	Semen	Bull	Supplementation of EVs isolated from bull seminal plasma in the extender for sperm freezing significantly improves the sperm cryostability	[136]

### Application of EVs in monitoring health status of dairy cattle

Liquid biopsy has emerged as a minimally-invasive, accurate and convenient method for disease diagnosis and prognostic monitoring. EVs are associated with the onset and progression of several diseases and physiological processes. And EVs are commonly present in most body fluids such as blood, milk, urine, amniotic fluid and semen [18]. The content and composition of EVs are influenced by physiological states and environmental factors, and thus the characterization of EVs can provide candidate biomarkers for the diagnosis of physiological states and diseases in dairy cattle. Furthermore, EV membranes contain lipid rafts, which confer high stability [137]. It was also demonstrated that the levels of proteins, markers and miRNAs in mEVs are not influenced after 7 days of storage at 4 °C [138]. Jiang et al. constructed a highly sensitive glycosyl-imprinted electrochemiluminescence (ECL) sensor capable of recognizing and capturing highly glycosylated EVs through glycosyl-imprinted polymer film. This sensor generates ECL signals through CD63 aptamer-bipyridine ruthenium, enabling accurate differentiation of EV content between cancer patients and healthy controls [139]. This further highlights the feasibility of using EVs-derived biomarkers.

miRNAs serve as essential cargo within EVs, enabling EVs to carry out biological functions. Alterations in miRNA expression are linked to pathophysiological states in dairy cattle and are utilized as diagnostic biomarkers [51]. Liu et al. reported that the number and protein concentration of milk-derived EVs in estrous cows were lower than those in non-estrous cows and identified 133 miRNAs that were differentially expressed in milk-derived EVs. The levels of miR-664a, -409b and -29c were significantly elevated in milk-derived EVs from estrous cows. These miRNAs may serve as candidate biomarkers for estrus identification in dairy cows [123]. It has been demonstrated that miRNA profiles in milk-derived EVs are altered from cows with subclinical mastitis [87]. Cai et al. also identified 18 differentially expressed miRNAs in milk-derived EVs of healthy and mastitic cows, and bta-miR-223-3p was the most up-regulated miRNA [124]. Another study also indicated that miR-375 was significantly decreased in milk-derived EVs from cows with mastitis compared to healthy cows, which may induce up-regulation of negative regulators of the immune response (e.g., IL7R, IHH, CTLA4, and IRF1), potentially contributing to the progression of mastitis [125]. Therefore, bta-miR-223-3p and miR-375 are suggested as candidate markers for mastitis identification. Milk is easily accessible and abundantly available, and these studies highlight the potential of milk-derived EVs in monitoring health status in dairy cattle.

The placenta releases EVs into the maternal circulation and circulating EVs may also provide a variety of information about the state of pregnancy and embryonic development [140]. For example, Zhao et al. found that bta-miR-499 was significantly elevated in the plasma of placenta-derived EVs from cows during early pregnancy. Further experiments demonstrated that bta-miR-499 increased the expression of bta-let-7 miRNA by targeting Lin28B, thereby suppressing the inflammatory response in bovine EECs [126]. This finding provides a novel approach for immune regulation at the maternal–fetal interface during early pregnancy and for early pregnancy diagnosis. Pohler et al. also studied circulating EVs in cows with different embryonic mortality and gestational status and found that the relative abundances of miR-25, -16b and -3596 were significantly increased in cows exhibited embryonic mortality compared to pregnant cows [127], indicating that these miRNAs could serve as potential biomarkers for monitoring the developmental status of early embryo. In addition, Chauhan et al. also reported that the level of bta-miR-195 in seminal plasma was related to fertility status and can be used as a potential marker for selecting high-fertility bulls in artificial breeding [58].

Hailay et al. reported that follicular fluid-derived EVs have differential miRNA expression profiles in always-positive cows (APCs) and always-negative cows (ANCs), and most of the differentially expressed miRNAs were up-regulated in the APCs and down-regulated in the ANCs [47]. Target prediction of five miRNAs (bta-miR-2285, bta-miR-451, bta-miR-132, bta-miR-486, bta-miR-874) whose expressions were significantly reduced in ANCs revealed their involvement in various pathways related to ovarian function, including apoptosis, cell cycle, TGF-beta signaling, hippo signaling, and others. Postpartal negative energy balance (NEB) is one of the main causes of fertility decline in dairy cows [141]. Thus, follicular fluid-derived EVs may provide information on their metabolic status and potentially help explain the mechanism causing reduced fertility rates in cows experiencing prolonged NEB.

Protein cargoes in EVs are linked to certain biological processes, indicating that EVs are also a potential source of protein biomarkers. Thus, Piibor et al. compared the proteomic profiles of uterine fluid-derived EVs from healthy cows and cows with subclinical endometritis (SE). They identified 248 differentially expressed proteins between the groups, but differential enrichment analyses revealed that only HtrA serine peptidase 1 (HTRA1) was significantly enriched in SE cows. This finding suggests that HTRA1 could be a potential diagnostic marker for SE [85]. Turner et al. [37] established a predictive modeling of fertility status by combining the proteomics of small EVs from two different cohorts of young and aged dairy cows with the different heritabilities and known pregnancy outcomes. This study was conducted in a larger cohort with methodological refinements to the EV enrichment strategy to enhance the reproducibility of the results. MINT sPLS-DA analyses demonstrated that the EVs predictive model exhibited better performance in predicting fertility status compared to plasma. It was also suggested that DNAI4, BAZ1A and HEXB could be candidate biomarkers for high-fertility cows, and HSPA4, MYOM2, WFIKKN2 could be candidate biomarkers for low-fertility cattle in the small EVs [37]. Because the EVs circulate widely and are relatively easy to collect, plasma small EVs could more accurately predict fertility phenotypes. As such, the development of fertility biomarkers in small EVs could be useful for improving the early fertility assessment, timely breeding and conception, and, thus, promote genetic improvement in dairy cattle.

By monitoring changes in the concentration and composition of EVs in serum, milk, and various body fluids, we can characterize the metabolic status, physiological cycle, disease development, and reproductive capacity of dairy cattle [142]. This would be helpful in developing individualized management plans to improve the economic efficiency of dairy farms. However, research on EVs-related biomarkers is still in its infancy and the isolation methods and analytical tools for EVs need to be further optimized.

### Application of EVs in the treatment of diseases in dairy cattle

The EVs are abundant and have high cell membrane tolerance and low immunotoxicity. EVs are capable of traversing diverse biological barriers, facilitating the exchange of proteins and genetic material between cells. Furthermore, their uptake exhibits cell-specificity [143]. Therefore, the EVs display great potential for application in the treatment of immune diseases, inflammatory diseases and optimization of drug delivery systems in dairy cattle [144, 145]. For example, Ghai et al. demonstrated that the administration of EVs prepared from bovine umbilical cord blood mesenchymal stem cells alleviated subclinical mastitis in dairy cows by upregulating angiopoietin-1, anti-inflammatory cytokines (IL-10) and anti-microbial peptides (LCN2, CST3, CATHL4). EVs administered either intravenously or locally demonstrate greater efficacy than antibiotics. They also effectively address key limitations of antibiotics, such as the inability to resolve infections within the 15-day window and antibiotic residues, providing a feasible and effective therapy for alleviating mastitis at subclinical stage [128]. In a study of cows with endometritis, miR-218 level was significantly reduced in exosomes derived from EECs and further impaired placental development by targeting secretion of frizzled related protein 2 [101].

Bovine colostrum-derived EVs have been reported to have potential anti-inflammatory and antioxidant properties [146]. Mecocci et al. isolated colostrum-derived EVs from Piedmontese cows within 24 h of calving and assessed their antimicrobial activity using an in vitro model of calf diarrhea. Pretreatment with colostrum-derived EVs exhibited anti-inflammatory via downregulating *TLR5*, *STING*, and *CGAS* in cells, as well as reducing adhesion of *Escherichia coli* [129]. Xiong et al. found that treatment with bovine colostrum-derived EVs could reduce the expression of pro-inflammatory factors in macrophages and protected tight junction protein expression, blood–milk barrier integrity and milk production by inhibiting the NF-κB signaling pathway, which is expected to be a therapeutic approach for mastitis [147]. Liang et al. demonstrated that treatment with EVs derived from colostrum, mature milk and mastitis milk alleviated *Klebsiella pneumoniae*-induced oxidative stress and ferroptosis in bovine mammary epithelial cells. This effect was mediated by upregulating the expression of KEAP1, HO-1, GPX4, S100A4, and ACSL4, which subsequently reduced mammary inflammation and restored the cellular barrier [91]. While no studies have investigated the use of milk-derived EVs for treating infectious diseases in dairy cattle by feeding, Liang et al. demonstrated that porcine colostrum-derived EVs could effectively alleviate intestinal villous damage, diarrhea, and reduce mortality in piglets infected with porcine epidemic diarrhea virus by inhibiting virus replication [148]. Moreover, milk-derived EVs exhibit resistance to degradation under conditions such as freezing and thawing, low pH and digestive enzyme, owing to their lipid bilayer membrane structure [30, 137]. These findings demonstrate the feasibility and efficacy of milk-derived EVs administered via feeding, implying that milk-derived EVs may serve as natural antimicrobial agents for the prevention and treatment of infectious diseases in dairy cattle.

Milk-derived EVs exhibit high stability, and the abundant yield of milk facilitates their large-scale commercial production [149]. Consequently, milk-derived EVs hold significant potential as drug carrier, offering a broad range of applications in the medical field. Xu et al. successfully developed a drug system of milk-derived EVs loaded with isobavachalcone and polymyxin B which significantly increased antimicrobial activity and accelerated wound healing by enhancing cell membrane erosion [130]. Milk-derived EVs, as natural and safe drug delivery carrier, require further optimization to enhance their efficiency and reduce the isolation and purification costs. In addition, Qu et al. found that milk-derived EVs functionalized with phosphatidylserine significantly increased the solubility and antimicrobial efficiency of α-mangostin and were capable of stable and sustained release in the gastrointestinal tract [131]. Oliver et al. also demonstrated that milk-derived EVs remain stable after gastrointestinal treatment simulated with digestive enzymes and bile [30]. This implies that EVs, as drug carriers, not only improve the solubility and efficacy of drugs, but also be used orally for drug delivery, providing a significant advantage over traditional intravenous administration. However, EVs are cleared by macrophages, resulting in a shorter half-life of treatment [150]. There are challenges in developing EVs-drug loading systems that avoid macrophage clearance and in maximizing the effects of vesicular drug delivery. This could be a fertile area of future research.

There is some evidence for EVs as suitable for vaccine delivery. Saenz et al. identified key pathogenic and immune-evading proteins in EVs, such as gamma-hemolysin, Sortase A and elastin-binding, by assessment of the molecular response and proteomic changes of EVs derived from mastitis-associated strains (*S. aureus*) on bovine monocyte-derived macrophages [89]. Bacterial-derived EVs are enriched with toxins and cell surface proteins that elicit immune responses, highlighting their potential as vaccine candidates. A study in mice demonstrated that immune cell-derived EVs could evade clearance by the immune system, thereby prolonging their therapeutic effect [151]. The application of molecular engineering techniques to overexpress certain antigens to enhance the efficiency of the immune response triggered by bacterial-derived EVs highlight the use of bacterial-derived EVs as vaccine candidates [152].

Because EVs are key mediators of bidirectional communication between microorganisms and hosts, they seem ideally suited for maintaining cattle health [153]. For instance, Zhou et al. reported that feeding mEVs induced changes in the gut microbiota of mice, and a similar approach if deemed effective could be used to inhibit the colonization of harmful bacteria in the gastrointestinal tract of dairy cows [154]. Manipulating the microbiota of dairy cattle through EVs may reduce the side effects associated with direct-fed microbials, such as overstimulation of the immune response, harmful metabolic reactions, and drug incompatibility [155–157]. However, the mechanisms of EVs-microbe interactions remain poorly understood. It is also unclear whether EVs influence gene expression and metabolic responses of the recipient animals by altering the gut microbiome. This could represent a fertile area for future research.

### Application of EVs in assisted reproduction

Progressive research on function and application of EVs has effectively promoted the development of assisted reproduction technology in dairy cattle. For instance, intraovarian injection of bovine mesenchymal stromal cells-derived exosomes induced proliferation and migration of ovarian stromal cells, thereby treating ovarian damage caused by ovum pick-up [132]. Stem cell-derived EVs may also provide a new strategy for the treatment of reproductive disorders such as postpartum anestrus and prolonged estrous intervals in heifers. Mitchell et al. demonstrated that milk-derived EVs loaded with miR-143 reduced prostaglandin E_2_ production in bovine EECs by suppressing cyclooxygenase, thereby supporting pregnancy establishment and maintenance [104]. Menjivar et al. showed that EVs isolated from bovine granulosa cell culture medium mitigated the adverse effects of heat stress on oocytes by enhancing mitochondrial function and regulating stress-related gene expression [133]. Benedetti et al. also identified bta-miR-34c as the most abundant miRNA in follicular fluid-derived EVs from receptive oocytes. Supplementation with miR-34c mimics improved blastocyst quality during in vitro maturation, further supporting the regulatory role of miR-34c in oocyte function and embryo development [46]. In addition, Hasan et al. indicated that EVs derived from follicular fluid of bovine large, medium and small follicles significantly improved sperm viability, capacitation and acrosome reaction [134]. This also suggests the feasibility of large-scale collection of follicular fluid EVs for commercialization, offering potential applications in optimizing gamete culture system and improving the quality of in vitro embryo culture.

Gad et al. reported that superovulation altered the miRNA abundance in plasma EVs of heifers. miR-199a-3p in EVs was upregulated after the superstimulatory response, whereas miR-17-5p and miR-182 were downregulated, which provided two potential biomarkers for predicting superstimulatory responses in heifers [135]. Qiao et al. reported that EVs derived from the bovine luteal phase uterus inhibited apoptosis by downregulating *BAX*, *BIP*, and *HSP70*, improving somatic cell nuclear transfer embryo culture conditions, which in turn increased blastocyst formation, hatching rate, blastocyst quality and structural integrity [53]. Another study indicated that miR-148b in oviductal fluid-derived EVs can be taken up by embryos and activate SMAD5-dependent BMP signaling to regulate cell proliferation and differentiation in embryos[97]. In addition, Kowalczyk et al. demonstrated that supplementation of EVs isolated from bull seminal plasma in the extender for sperm freezing significantly improves the sperm cryostability by protecting the sperm mitochondrial and cytoplasmic membranes [136].

### Techniques for application of EVs in practice

EVs have great potential for early clinical diagnosis and disease treatment, thus, it is critical to develop new technologies to analyze and use EVs [158]. Silva et al. compared the novel high-resolution single-vesicle analysis methods Nanoflow cytometry, Single-Molecule Localization Microscopy and ExoView, and concluded that Single-Molecule Localization Microscopy is currently the most accurate technique to quantify EVs at a single‐vesicle and single-molecule level [159]. Kim et al. compared the characterization of EVs using commercial high-sensitivity flow cytometers (CellStream/CytoFLEX) and a custom single-molecule flow cytometer (SMFC). They found that antibody copy numbers for EVs exhibited good agreement after applying data filtering to results from commercial instruments and SMFC [160]. In addition, signals of EVs in body fluids are weak, amplification of the signal is needed to detect EVs in the early stages of the disease, including enzyme-free and enzyme-assisted amplification, fluorescence-based amplification and electrochemiluminescence-based amplification [161].

The use of miRNA in EVs as diagnostic markers and commercial application necessitates careful consideration of EVs isolation method, miRNA extraction techniques, and sample types [162]. In addition, current miRNA detection technologies have limitations. RT-qPCR is commonly used in the initial research and discovery phase of biomarkers due to its high sensitivity and specificity for detecting miRNAs in EVs from body fluids, but it is unsuitable for clinical practice because of its complex and time-consuming operation. Biosensors based on optical fluorescence to detect changes in intrinsic physical parameters induced by miRNA molecules can detect target miRNAs in natural state. This method is not only more rapid, reliable and cost-effective, but also has low requirements for sample volume and detection limit [163]. For instance, Laser tweezers Raman spectroscopy (LTRS) allows the characterization of individual EVs signals in a physiological setting by restricting background signal interference. This method enables rapid and accurate analysis of individual EVs signals from bulk EVs, identifying marker molecules within specific captured EVs, which highlights its significant potential for commercial applications [164]. Single Molecule array technology (SIMOA) is an automated and easy-to-use immunoassay for EVs. For example, Wei et al. employed SIMOA technology to develop two different EV detection kits (Epcam-CD63 and CD9-CD63) targeting universal EVs and tumor-EVs, respectively [165]. This method shows exceptional performance in tumor diagnosis and provides a reliable approach for detecting protein biomarkers in EVs.

The primary challenge in enabling the commercial application of EVs is achieving the large-scale purification. Milk, which is rich in EVs, represents a scalable, safe and economical source. However, soluble proteins in milk can impede EVs purification, necessitating pre-treatment. Benmoussa et al. found that sodium citrate (1% final) could disrupt casein micelles in milk, thereby enhancing the purification efficiency of milk-derived EVs [166]. Other studies have also indicated that single purification methods are insufficient for isolating milk-derived EVs. Effective isolation requires pretreatment (e.g., acid, calcium chelator, or chymosin), ultracentrifugation and combination with techniques such as size exclusion, microfluidic technologies, or well-established commercial Core700 to achieve large-scale and high-purity EVs isolation [167–170].

Furthermore, the optimization of EVs delivery and release techniques would need to be considered simultaneously [171]. Han et al. developed a strategy to incorporate EVs coated with oxygen nanobubbles into a self-healing hydrogel matrix as wound dressings, thus, providing a method for the efficient cargo delivery of EVs under wound hypoxic conditions [172]. Yang et al. constructed a sustained-release system of EVs using polydopamine functionalized 3D printing gelatin/hyaluronic acid/nano-hydroxyapatite scaffolds and verified that this system loaded with EVs secreted by bone marrow mesenchymal stem cells had better promotion of bone regeneration in the calvarial defect model of diabetic rats [173]. Ilahibaks et al. explored an endogenous EV protein delivery approach that utilizes cellular processes to produce and incorporate biotherapeutic payloads into EVs [174]. Nie et al. fabricated a bioadhesive EVs microcarrier, via applying microfluidic technology to encapsulate EVs derived from IL-27-carrying MSC in an adhesive hydrogel with DMA and GelMA, and administered them rectally to inflammatory bowel disease rats [175]. They observed that these microcarriers could be targeted to adhere to the colon surface, reduce inflammation and repair the intestinal barrier, all of which are more efficacious than traditional intravenous injections. These data suggest that rapid advancements in technologies for the characterization, analysis and delivery of EVs offer promising strategies for the applications of EVs in dairy cattle. Jing et al. demonstrated that fusing a cell-penetrating peptide with green fluorescent protein enabled efficient loading of proteins and peptides into EVs. Furthermore, oral administration of milk-derived EVs loaded with an anti-TNF-α nanobody and the antimicrobial peptide LL37 significantly alleviated ulcerative colitis in mice [176]. Chen et al. developed an “ultrasonication and extrusion-assisted active loading” method, achieving a drug-encapsulation efficiency approximately tenfold higher than passive loading [177]. This evidence suggests that rapid advancements in EVs manufacturing technologies will further enhance the feasibility of large-scale applications of EVs for disease monitoring and treatment in dairy cattle.

## Prospects and limitations

There is substantial data indicating that EVs, widely secreted by cells, are important vehicles for mediating host health [178]. These structures can be efficiently taken up by cells and deliver their cargo to elicit specific physiological responses. Due to the low immunogenicity, EVs hold great promise for research as drug delivery vehicles. However, there are still limitations in terms of clinical applications. At present, the isolation and large-scale production of EVs remain challenging. General EV isolation techniques include ultracentrifugation (dUC), size exclusion chromatography (SEC) and ultrafiltration (UF) [179]. dUC is suitable for large-scale isolation of EVs, but does not allow the separation of different types of EVs [180]. SEC prevents protein contamination, but column capacity is limited [181]. UF has relatively less separation time and cost, but is prone to membrane clogging. Tangential flow filtration can be adopted to solve the pore clogging, but EVs may be damaged [182]. Other contaminants contained in EVs isolated by existing techniques may interfere with their quantification. Thus, further optimization of the isolation and purification procedures is needed.

Although it is theoretically possible to use dietary EVs to improve cattle health, prevent and treat disease, it is necessary to further clarify the relationship between EV cargo composition and the physiological response by cattle. In addition, if intestinal delivery is an important goal, there needs to be a greater understanding of the degree of ruminal metabolism and intestinal bioavailability of dietary EVs. The function of EV cargo and the role of EVs in the regulation of the host gut microenvironment also need to be further explored. In addition, the quantification of EVs needs to be further standardized and clarified including the use of one parameter or a combination of parameters such as particle number, RNA, protein amount, and lipid abundance among the most important. More studies are needed to determine the effective doses of EVs that can trigger a positive physiological response in the animal.

Various analytical tools for EVs have been developed, such as the use of surface plasmon resonance sensors to capture EVs and combine kinetics, fluorescence, antibodies and aptamers for EVs to quantify EVs and detect the composition of EVs [183]. The potential of surface plasmon resonance sensors for EVs analysis has also been validated in clinical studies for cancer [184], Alzheimer’s disease [185] and cardiovascular diseases [186]. In the future, it is envisioned that sensors will be developed to capture EVs in dairy cattle. Such data can then be analyzed to determine individual health status and genetic differences, both of which can be used for adjustments in management strategies and breeding selection. However, before these potential applications can take place, protocols for standardization of EV sampling, cell source, isolation and analysis techniques need to be developed and validated.

The heterogeneity of methods leads to differences in results across studies, and the data are difficult to analyze in a comparative manner. Larger-scale studies are still needed to expand the database of EVs under different conditions and contexts, establish standardized guidelines for use in laboratory studies, and increase the reliability and rigor of research in the field of EVs. Furthermore, the volume required for characterization and analysis is relatively large, resulting in a waste of EVs. Thus, developing and improving vesicle characterization techniques are urgent undertakings.

## Conclusion

EVs serve as crucial mediators for intercellular communication by transporting bioactive molecules. This review summarizes research progress on EVs in dairy cattle (mammary, gastrointestinal tract, blood, uterus, ovary, embryo and semen) and their regulatory mechanisms in metabolism, immunity, reproduction, mammary gland development, and intestinal function. EV composition reflects the physiological and pathological states of their parent cells. We discussed their applications in health monitoring, disease treatment and assisted reproduction in dairy cattle. Furthermore, we evaluate the commercial potential and current limitations of EVs. This understanding of EV roles and mechanisms in dairy cattle may enable development of novel biomarkers and therapeutic strategies, ultimately enhancing dairy cattle health, productivity and economic outcomes.

## Data Availability

Not applicable.

## Abbreviations

AI: Artificial intelligence
*A*. *muciniphila*: *Akkermansia muciniphila*
ANCs: Always-negative cows
APCs: Always-positive cows
AQP2: Aquaporin-2
BLV: Bovine leukemia virus
Bt: Bacteroides thetaiotaomicron
dUC: Differential ultracentrifugation
EBL: Enzootic bovine leukosis
ECL: Electrochemiluminescence
EECs: Endometrial epithelial cells
EVs: Extracellular vesicles
GO: Gene Ontology
HMGB1: High mobility group box 1
*H. pylori*: *Helicobacter pylori*
*H*.* somni*: *Histophilus somni*
HTRA1: HtrA serine peptidase 1
IFNT: Interferon tau
IL: Interleukin
KEGG: Kyoto Encyclopedia of Genes and Genomes
MAC-T: Bovine mammary epithelial cell lines
MFG-E8: Milk fat globule EGF factor 8
miRNAs: MicroRNAs
NEB: Negative energy balance
*S*.* aureus*: *Staphylococcus aureus*
SEC: Size exclusion chromatography
SPHINX: Slow Progressive Hidden INfections of variable latency
UF: Ultrafiltration
